# Rhino-orbital Mucormycosis as a complication of severe COVID-19 pneumonia

**DOI:** 10.1016/j.idcr.2021.e01293

**Published:** 2021-09-25

**Authors:** Mohammed A. Alamin, Mohammed Abdulgayoom, Sushil Niraula, Elabbass Abdelmahmuod, Ashraf O. Ahmed, Mohammed I. Danjuma

**Affiliations:** Hamad General Hospital, Internal Medicine Department, Doha, Qatar

**Keywords:** COVID-19, Mucormycosis, SARS-CoV2, Rhizopus, Black Fungus

## Abstract

Mucormycosis has multiple clinical phenotypes, which are more common in immunocompromised patients, especially those with diabetes mellitus. Debilitating rhino-orbital-cerebral and pulmonary infections by far represent the most typical clinical phenotypes associated with these fungi. Mucormycosis is an uncommon infection; however, there have been isolated sporadic tiny outbreaks around the world. With the substantial increase in COVID-19 cases in India, there is a parallel increase in the number of cases of Mucormycosis. A few reports raising unusual concomitant mucormycosis in COVID-19 patients have raised a possible association between the two diseases.

We report a 59-year-old male with an established history of uncontrolled diabetes mellitus admitted to the hospital with severe COVID-19 pneumonia (severity ascertained according to WHO classification) treated with steroids and discharged home following full recovery. However, one week later, he presented with right eye ophthalmoplegia and complete loss of vision, which was subsequently established as orbital Mucormycosis.

This case highlights the need for heightened awareness of this atypical secondary infection (especially systemic mycosis) in patients recovering from COVID-19 infection.

## Introduction

Mucormycosis or Zygomycosis is a term that represents different syndromes of fungal infection, most commonly manifesting as a severe form of rhino-orbital-cerebral or pulmonary infections [1]. Diabetes Mellitus and immunocompromised patients are most commonly affected [2]. With the emerging COVID-19 pandemic, some cases were reported suggesting a possible association between SARS-Cov2 infection and development of mucormycosis; currently suggested to be possibly due to COVID-19 infection or its treatment (steroids or tocilizumab) [3].

Herein we report a 59 years old male with a known history of diabetes who was treated with steroids and lopinavir/ritonavir for severe covid-19 infection. He presented one week after with right eye ophthalmoplegia and complete loss of vision and was found to have mucormycosis infection due to Rhizopus species. At the time of presentation, the patient was hyperglycemic with no evidence of ketoacidosis.

## Case presentation

A 59-Year-old male whose past medical history was significant for type 2 diabetes mellitus (on lifestyle modification) and transsphenoidal surgery in 2019 for symptomatic pituitary adenoma presented with symptomatology consistent with severe covid pneumonia. He was treated with a drug cocktail including dexamethasone and Lopinavir/Ritonavir for five days and subsequently discharged after complete recovery. Prior to this, the patient was not on any long-term medications (notably, he was not on steroids considering his history of transsphenoidal surgery).

**Fig. 1:**
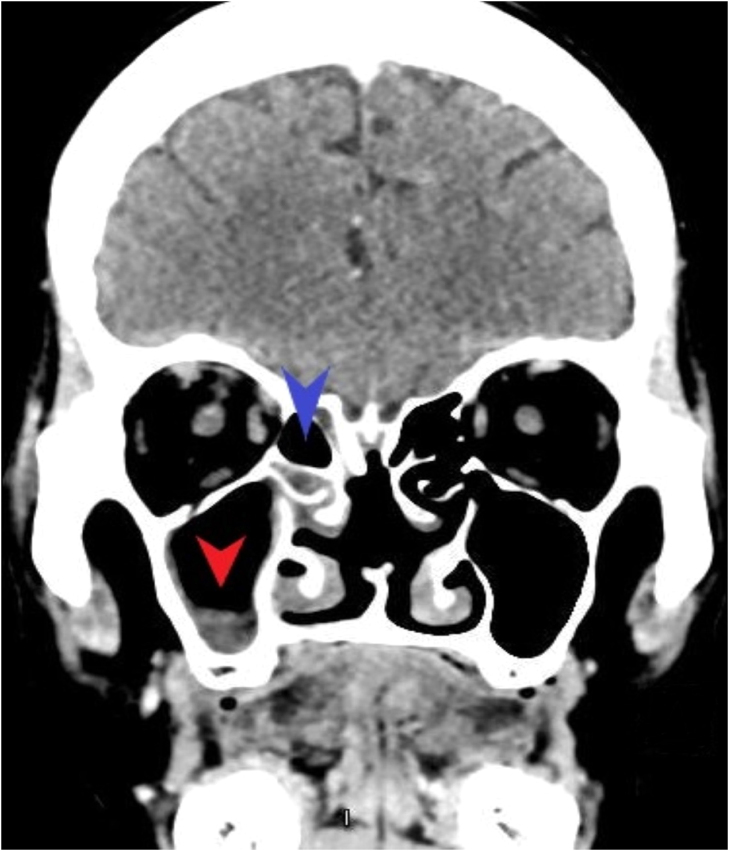
C.T. Head scan with contrast (coronal view) showing partial opacification of the right ethmoid (blue arrow-head) and right maxillary (red arrow-head) sinuses. CT: Computed Tomography.

**Fig. 2:**
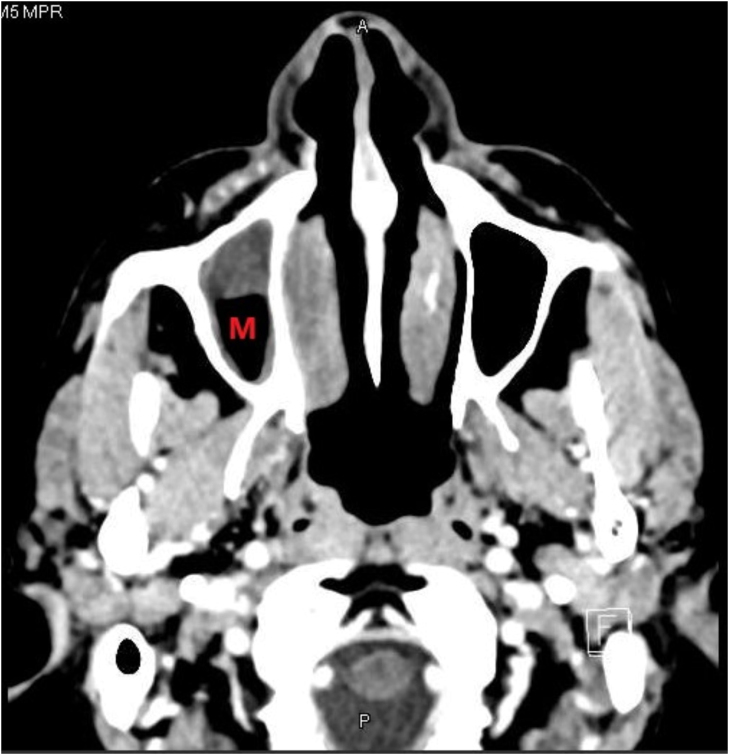
C.T. Head with contrast showing right maxillary sinus opacification (red "M" letter). CT: Computed Tomography.

**Fig. 3:**
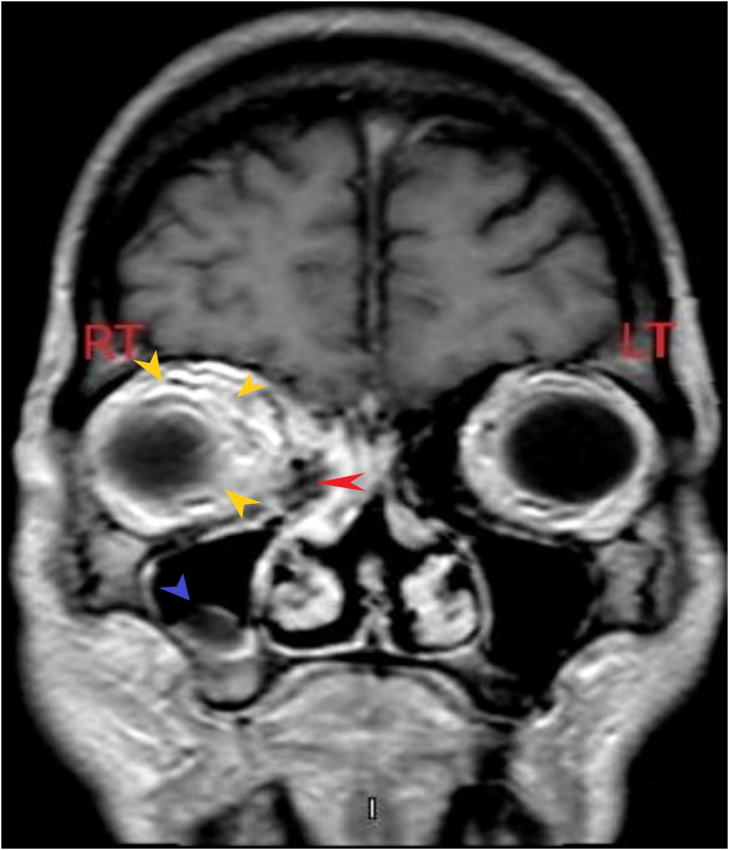
MRI Head with contrast (T1 weighted coronal view) scan showing enhancement at the right periorbital area (yellow arrow-heads) suggestive of periorbital cellulitis, along with opacification at right ethmoidal (red arrow-head) and right maxillary (blue arrow-head) sinuses. MRI: Magnetic Resonance Imaging. R.T.: Right, L.T.: Left.

One week after discharge, the patient presented with severe right eye pain, ptosis, and complete loss of vision. On examination, the patient had right-sided total ophthalmoplegia with complete paralysis of extra and intraocular muscles, right eye ptosis (with dilated fixed pupil), and severe pain and tenderness around the periorbital area. On admission, the patient had hyperglycemia (random blood glucose was 20.3 mmol/L) with no evidence of ketoacidosis. C.T. sinuses scan did show partial opacification of the right ethmoid, maxillary, and right frontal sinuses by mucosal thickening and retained secretions (Fig. 1, Fig. 2). Subsequent MRI head and orbit scan showed features suggestive of right orbital pre and post-septal cellulitis (Fig. 3). The patient was commenced on ceftriaxone, vancomycin, and metronidazole, and adjuvant steroids (for possible Tolosa-hunt syndrome). Further into his admission, vancomycin and steroids were discontinued, and anidulafungin was added empirically; however, the patient's symptoms did not improve.

Functional endoscopic sinus surgery was done, which showed polypoidal thickening of the right ethmoid sinuses, no pus or blackish discoloration, with no signs of acute infection. In addition, a tissue culture grew Rhizopus species, and blood cultures grew Microbacterium aurum.

The patient remained symptomatic and was commenced on hyperbaric oxygen sessions and had his antimicrobial regimen changed to liposomal amphotericin B and later to posaconazole (as the patient developed amphotericin-induced acute kidney injury); no surgical intervention was contemplated. The patient received four sessions of hyperbaric oxygen therapy as an inpatient, with some improvement in his pain. He was subsequently discharged with ophthalmology and ENT outpatient follow-ups and scheduled eye enucleation for total eye blindness.

## Discussion

Mucormycosis is emerging as one of the morbidities and mortality-prone complications of covid-19 infection, often in immunocompromised patients and those with type 2 diabetes. This mycosis often presents as debilitating rhino-orbital-cerebral and pulmonary infections [4]. Mucorales organisms are common in nature and can be found on decaying plants and in the soil. Rhizopus, Mucor, and Rhizomucor are the most common species in human infections [5]. Infarction and necrosis of host tissues are hallmarks of mucormycosis, often attributed to hyphae invasion of the vasculature [6]. Rhino-orbital-cerebral mucormycosis is presumed to start with inhalation of spores into the paranasal sinuses of a susceptible host. In contrast, in pulmonary mucormycosis, inhalation of spores into the bronchioles and alveoli is presumed to be the initial trigger [7].

The exact incidence of mucormycosis is difficult to be estimated as it is an underreported disease, and the risk varies in different populations [8]. A study of 929 instances of mucormycosis recorded between 1940 and 2003 showed that diabetes mellitus was the most prevalent risk factor, accounting for 36% of cases, followed by hematologic malignancies (17%) and solid organ or hematopoietic cell transplantation (12%) [9].

The mainstay of therapy for mucormycosis is a combination of surgical debridement of affected tissues and antifungal medication; this is besides eliminating its predisposing factors, such as neutropenia, hyperglycemia, and metabolic acidosis. Some individuals with mucormycosis have received hyperbaric oxygen therapy, although the effectiveness of this treatment has yet to be determined [10]. Interestingly, our patient had hyperbaric oxygen therapy with unclear clinical outcomes as his pain and visual loss failed to improve.

Mucormycosis is an uncommon infection; however, there have been isolated instances and tiny outbreaks worldwide. Along with the substantial increase in COVID-19 cases in India, mucormycosis cases have also increased. Alongside, with few case reports of mucormycosis infection in COVID-19 patients, this has raised the possible association between the two diseases [11].

Since the initial reports of coronavirus disease 2019 (COVID-19) and the discovery of the new coronavirus that causes it, severe acute respiratory syndrome coronavirus 2 (SARS-CoV-2), the infection have grown to encompass more than 150 million confirmed cases globally [12]. Glucocorticoids are affordable and widely available and thus far have been established to be amongst a handful of therapies with proven reduced morbidity and mortality benefit in Hypoxemic patients with COVID-19 clinical syndrome. However, it can increase the risk of secondary infection, hyperglycemia, and DKA, especially in patients with underlying undiagnosed Diabetic Mellitus [13]. Its indiscriminate use in the "second wave" of the COVID-19 pandemic, for example, may probably have accounted for the unusually high proportion of case reports of mucormycosis associated with COVID-19 coming from the Indian sub-continent. Suffice it to say that this is still under ongoing epidemiological review. Another drug increasingly used in COVID pneumonia is tocilizumab, which is also linked to an increased risk of secondary infection [14]. Though to our knowledge, no report or study has thus far linked it with mucormycosis.

COVID 19 infection has also unmasked many underlying undiagnosed immunocompromising conditions like diabetes mellitus and leukemia [15].

As our knowledge about the biology of COVID-19 and its clinical correlates/therapeutics increase, other explanation for the association between COVID 19 infection and mucormycosis may emerge, but for now, it could be either due to COVID treatment (direct effect or by inducing hyperglycemia and DKA) and/or due to the underline immunocompromising conditions that have been unmasked by the COVID 19. Patients with acute proptosis, an increased intraocular pressure, rapid visual loss, ophthalmoplegia, fixed dilated pupil, or afferent pupillary defect should be suspected of an undiagnosed mucormycosis [16]. Mucormycosis carries a high mortality rate, making early diagnosis and prompt treatment a sine-qua-non to prevent catastrophic outcomes [17].

Our patient appears to have all the risk factors thus far established to predispose to mucormycosis, including COVID 19 pneumonia, uncontrolled diabetes mellitus, receiving steroids for COVID 19, and the history of transnasal transsphenoidal surgery.

## Conclusion

A wide range of secondary infections and even opportunistic infections can complicate COVID 19 course. Early diagnosis and prompt treatment are essential to improve the outcome in mucormycosis, so high clinical suspicion is required from physicians and ophthalmologists involved in the care of COVID patients (especially those with rhino-orbital-cerebral lesions).

## Funding

Open access funding provided by the Qatar National Library.

## Ethics approval and consent to participate

Written informed consent was obtained from the patient for publication of this case report and any accompanying images. This case report was approved by Hamad Medical Corporation's Medical Research Center under number MRC-04–21–606.

## CRediT authorship contribution statement

**Mohammed A. Alamin:** Writing, Editing, Literature review, Correspondence, Final approval. **Mohammed Abdulgayoom:** Writing, Editing, Literature review, Final approval. **Sushil Niraula:** Editing, Final approval. **Elabbas Abdelmahmuod:** Editing, Final approval. **Ashraf O. Ahmed:** Imaging interpretation, Reporting, Final approval. **Mohammed Danjuma:** Editing, Final approval, Supervisor.
